# Relationships between Vacant Homes and Food Swamps: A Longitudinal Study of an Urban Food Environment

**DOI:** 10.3390/ijerph14111426

**Published:** 2017-11-21

**Authors:** Yeeli Mui, Jessica C. Jones-Smith, Rachel L. J. Thornton, Keshia Pollack Porter, Joel Gittelsohn

**Affiliations:** 1 Center for Human Nutrition, Department of International Health, Johns Hopkins Bloomberg School of Public Health, 615 N. Wolfe Street, Baltimore, MD 21205, USA; jgittel1@jhu.edu; 2 Department of Health Services & Nutritional Sciences Program, School of Public Health, University of Washington, Seattle, WA 98195, USA; jjoness@uw.edu; 3 Center for Child and Community Health Research, Division of General Pediatrics and Adolescent Medicine, Department of Pediatrics, Johns Hopkins School of Medicine, Baltimore, MD 21205, USA; rjohns21@jhmi.edu; 4 Department of Health Policy and Management, Institute for Health and Social Policy, Johns Hopkins Bloomberg School of Public Health, Baltimore, MD 21205, USA; kpollac1@jhu.edu; 5 Global Obesity Prevention Center (GOPC) at Johns Hopkins University, 615 N. Wolfe Street, Baltimore, MD 21205, USA

**Keywords:** food swamp, food environment, neighborhood, food store, vacant home, African American, low-SES

## Abstract

Research indicates that living in neighborhoods with high concentrations of boarded-up vacant homes is associated with premature mortality due to cancer and diabetes, but the mechanism for this relationship is unclear. Boarded-up housing may indirectly impact residents’ health by affecting their food environment. We evaluated the association between changes in vacancy rates and changes in the density of unhealthy food outlets as a proportion of all food outlets, termed the food swamp index, in Baltimore, MD (USA) from 2001 to 2012, using neighborhood fixed-effects linear regression models. Over the study period, the average food swamp index increased from 93.5 to 95.3 percentage points across all neighborhoods. Among non-African American neighborhoods, increases in the vacancy rate were associated with statistically significant decreases in the food swamp index (b = −0.38; 90% CI, −0.64 to −0.12; *p*-value: 0.015), after accounting for changes in neighborhood SES, racial diversity, and population size. A positive association was found among low-SES neighborhoods (b = 0.15; 90% CI, 0.037 to 0.27; *p*-value: 0.031). Vacant homes may influence the composition of food outlets in urban neighborhoods. Future research should further elucidate the mechanisms by which more distal, contextual factors, such as boarded-up vacant homes, may affect food choices and diet-related health outcomes.

## 1. Introduction

Neighborhood environments and resources may be important for eating behaviors and obesity risk [1,2,3,4]. Studies have examined differences in the distribution of food outlets by neighborhood socioeconomic status (SES) and racial composition, reporting that low-SES and predominantly African American neighborhoods tend to have fewer supermarkets, fruit and vegetable markets, and natural food stores, while having greater access to liquor stores and outlets that promote unhealthy eating, such as fast food restaurants and convenience stores, compared to high-SES and predominantly White neighborhoods [5,6,7]. This disproportionate exposure to unhealthy versus healthy food outlets, also known as food swamps, has recently gained attention by capturing the overall composition of food outlets available to consumers, versus focusing on one specific food outlet type at a time [8]. However, there is still limited understanding and empirical evidence of neighborhood-level risk factors that may influence where certain food outlets locate, therefore, contributing to food swamps in some urban neighborhoods and not in others.

The physical appearance of a neighborhood, including sidewalks, buildings, as well as the state of disrepair of these elements, such as cracked sidewalks or boarded up vacant homes are increasingly recognized as potentially influencing the well-being of communities and its residents [9,10]. One ecological study of 107 U.S. cities showed that vacant housing was positively associated with premature mortality due to cancer, diabetes, homicide, and suicide, after adjusting for sociodemographic factors [11]. Additionally, as part of the Framingham Heart Study, researchers reported that for each additional foreclosed property, which typically sit vacant, nearby residents experienced a statistically significant increase in body mass index (BMI) [12]. While vacant homes have been linked to adverse health outcomes and weight gain, the mechanism through which this occurs is poorly understood.

According to the ‘broken windows’ theory, the physical appearance of a neighborhood provides cues influencing perceptions of social norms in the area [13,14]. Specifically, the presence of boarded up vacant homes or broken windows may signal that the area is uncared for or that community members are unable to maintain an orderly social and physical environment. Further, such cues have been theorized to generate a sense of fear, deter social interactions, diminish trust among some community members, and increase opportunities for crime due to a perceived low risk of being caught [11,15]. For example, a qualitative study of residents in New York indicated that a declining neighborhood led to increased isolation and interfered with the community’s ability to organize and form relationships [16]. Similarly, business owners in neighborhoods of physical disrepair may also perceive declining disposable incomes and a lack of social capital. Because of these barriers to a more viable market, food outlets may prefer to locate in areas where establishing a business is perceived to be less of a risk [11]. Thus, only certain businesses may choose to remain or open in neighborhoods that have a large degree of boarded-up vacant homes. In this way, vacant homes, which itself is a product of economic decline, might play a role in determining the composition of food outlets in a given neighborhood.

**Figure 1:**
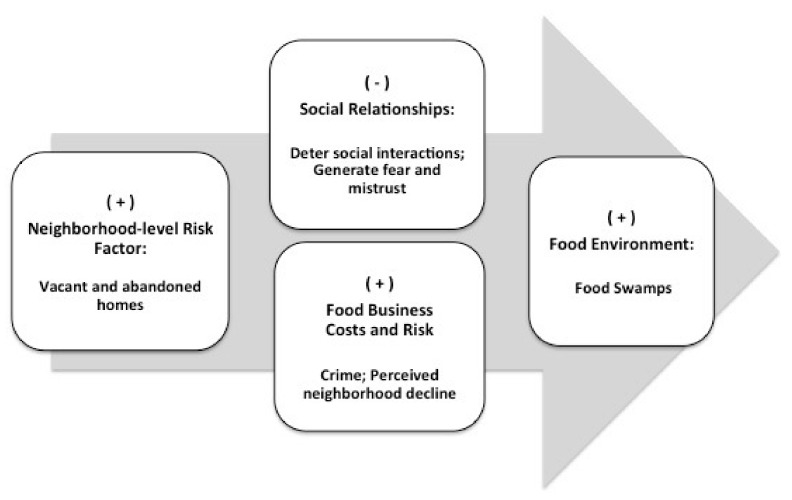
Simplified conceptual framework of proposed relationships between vacant and abandoned homes (proxy of neighborhood-level risk factor), social relationships among community members, decisions of food outlet owners related to business costs and perceived risk, and food swamps in Baltimore, MD, USA. A positive relationship is represented by ‘+’, and a negative relationship is represented by ‘−‘ (i.e., increases in neighborhood-level risk factors negatively impact social relationships, increases food business costs and perceived risk, and contributes to food swamps).

Building on a prior study that explored the influence of disrepair in the social environment, namely crime rates, on the neighborhood food environment [17], the current study draws from ‘broken windows’ theory and prior literature to explore the longitudinal relationships between vacant homes and the food environment in Baltimore, MD from 2001 to 2012 (Figure 1). Specifically, we investigated the following research questions: (1) What is the association between changes in vacancy rates and changes in the relative density of unhealthy food outlets as a proportion of all food outlets, which we termed the food swamp index? and (2) Does this association vary by neighborhood racial composition, SES, or population change? A more comprehensive understanding of the potential role of vacant homes in shaping the mix of outlets in the food environment may be useful as public health practitioners, city planners, and housing officials consider various options to create more health-promoting neighborhoods.

## 2. Materials and Methods

### 2.1. Study Region

The study area for this research was Baltimore, MD, an urban area of 80.9 square miles. Baltimore provides an ideal setting in which to explore the study’s research questions because shifts in employment opportunities from the city center to the suburbs and globally, followed by population and neighborhood change, was a phenomenon experienced in many cities across the U.S., including Baltimore [14,18,19]. As a result of significant job loss in the city, a population of nearly 1 million residents in the 1950’s is now a city of an estimated 621,000 people, with 24.2 percent of people living below the poverty line [20]. One of the negative consequences of this decline in population is the estimated 17,000 vacant homes in Baltimore today [21]. Moreover, as wealthier residents have left for the suburbs over the years, changes in neighborhood retail ensued. For example, by 2000, Baltimore City had experienced the closing of some major local supermarket chains, resulting in the loss of nearly 30 local supermarkets [22]. Conversely, the opposite trend has occurred in terms of smaller food outlets, with an overall increase in those that are known to predominantly sell unhealthy options, such as local corner stores, carryouts, fast food restaurants, and convenience stores [17]. As such, many Baltimore residents have access to relatively few options for healthy food outlets, while those that provide nutrient-poor, energy dense foods are plentiful [23,24,25,26,27].

**Figure 2:**
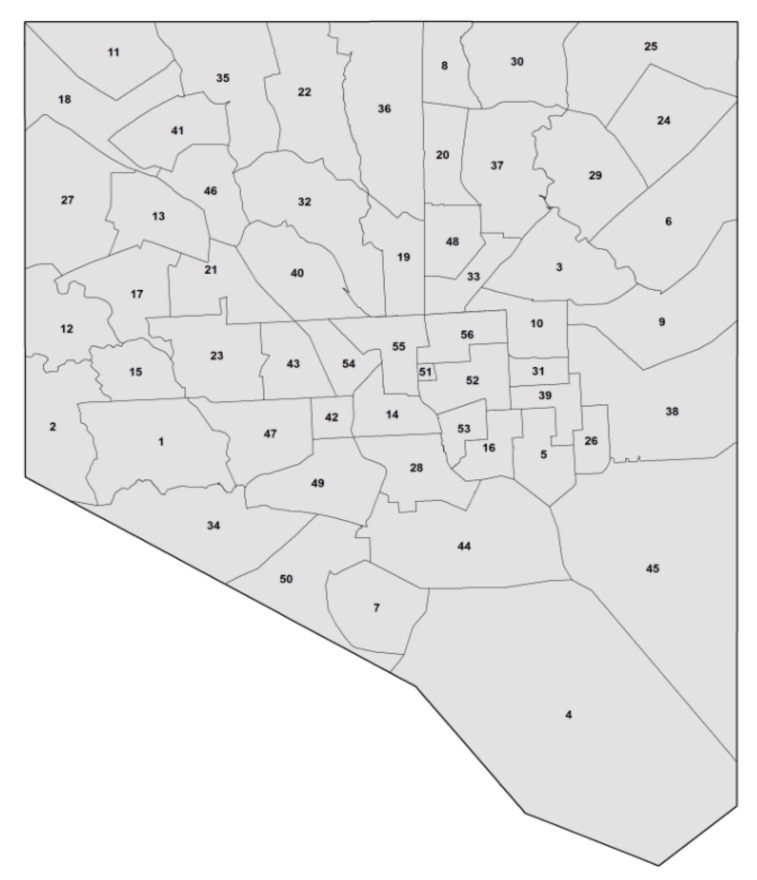
Community statistical areas (CSAs) in Baltimore, MD (n = 55). The Baltimore City Detention Center is identified as 51 but is not a designated CSA, therefore, is excluded from the CSA count [17].

Baltimore neighborhoods were assigned to 55 community statistical areas (CSAs) (Figure 2). CSAs were defined by the Baltimore City Data Collaborative and the City Department of Planning based on several criteria: (1) CSA boundaries must line up with boundaries of census tracts; (2) CSAs must comprise of 1–8 census tracts and include a population size between 5000 to 20,000 residents; (3) CSAs must be relatively homogenous in its demographics; and (4) CSAs must reflect community members’ perceptions of neighborhood boundaries, as understood by City planners. In 2010, census tract boundaries were modified, affecting two of the 55 CSAs: CSA 52 and CSA 53.

### 2.2. Dependent Variables

The dependent variable for this analysis was the food swamp index, which was selected as the primary outcome because of our interest in the change in the overall composition of food outlets over time. An adaptation of the Modified Retail Food Environment Index (mRFEI) [28] and Physical Food Environment Index (PFEI) [29], the food swamp index was quantified as the following for each CSA:

$$\frac{density\;of\;BMI\;unhealthy\;outlets\;+\;density\;of\;BMI\;intermediate\;outlets}{density\;of\;all\;food\;outlets}\;\times \;100$$

Dividing by the density (outlets per sq. mile) of all food outlets rather than using a ratio of unhealthy to healthy outlets prevented the occurrence of an undefined number and allowed us to include neighborhoods that had zero healthy outlets. Further, given prior research that has reported on the low nutritional quality and high energy density of food away from home (e.g., from full-service restaurants or other takeaway food stores) and our interest in exploring the over-concentration of outlets providing less healthy, energy-dense food options that inundate healthier food options, we included the BMI-unhealthy and BMI-intermediate outlets in the numerator of our food swamp index calculation [30,31]. There were no CSAs with zero food outlets.

We obtained and combined food environment data from two commercial databases, Dun & Bradstreet (D&B) and InfoUSA to identify, geocode, and classify food outlets in Baltimore from 2001 to 2012 [32,33,34,35]. As reported by prior validation studies, sensitivity is only 65 percent for InfoUSA and 55 percent for D&B when using one of these data sources alone, but increases to 81 percent when both data sources are combined [33]. Standard Industrial Classification (SIC) codes were used to identify the following food establishments: 53 (general merchandise stores), 54 (food stores), 5541 (gasoline service stations), 5812 (eating places, excluding drinking places with alcoholic beverages), and 5912 (drug stores and proprietary stores).

Using automated text searching in Stata/SE 14.1 (College Station, TX, USA) and manual review, each database was de-duplicated based on entry name and address. On average, approximately 8 percent of entries in the InfoUSA dataset and 7 percent of entries in the D&B dataset were deleted due to duplication. Next, the D&B data was appended to the InfoUSA data for each observation year, and another round of de-duplication was conducted based on entry name and addresses. In the appended dataset, about 27 percent of entries were removed due to duplication. We geocoded the street address for each outlet using ArcGIS 10.1 (Environmental Systems Research Institute: Redlands, CA, USA, 2012) and the Maryland Composite Locator service. The Maryland Composite Locator service, maintained by the Maryland Department of Information Technology, employs a number of individual locators, including address points, parcel points, and street centerlines to help find the best match for each address being geocoded. After the initial geocoding process was completed, we used the Interactive Rematch dialog box in ArcGIS to individually review and find addresses for each outlet with a matched tied address, using Google search and Google Street View. After the manual review and matching, we achieved a final average match rate of 99.8 percent across all years. We then removed all outlets located beyond Baltimore City boundaries and all duplicate entries.

To classify food outlets, we assigned each of 175 SIC definitions to one of 10 crude categories of food outlets: carry-out restaurants, fast food restaurants, full-service restaurants, convenience stores, small grocers/corner stores, superstores, general merchandise stores, healthy specialty stores, mixed specialty stores, or unhealthy specialty stores (Table S1). We implemented additional procedures to minimize misclassification of outlets over the study period by tagging outlets, based on keywords (Table S2), a list of top U.S. fast food restaurants (Table S3), a previously validated list of Baltimore food outlets [23], and consultation with local experts. Using a food outlet classification scheme similar to Rundle et al., the 10 crude categories were then further collapsed into 3 composite categories: BMI-healthy outlets, BMI-intermediate outlets, and BMI-unhealthy outlets [36]. BMI-healthy outlets were considered those that offer a larger range of fresh, generally more affordable, healthy foods; BMI-unhealthy outlets were considered those that offer predominantly unhealthy foods; and BMI-intermediate outlets were considered those whose classification into ‘healthy’ or ‘unhealthy’ was uncertain [36]. The BMI-healthy outlets composite category included: healthy specialty stores (e.g., fruit and vegetable markets, fish and seafood market), superstores (e.g., wholesale clubs), and supermarkets. The BMI-intermediate outlets composite category included full-service restaurants and mixed-specialty stores (e.g., gourmet food stores, juice shops). The BMI-unhealthy outlets composite category included carry-out restaurants, fast food chain restaurants, convenience stores, small grocers/corner stores, unhealthy specialty stores (e.g., candy stores, ice cream parlors), dollar stores, pharmacy chain stores, and gas station chains.

### 2.3. Independent Variables

The independent variable of interest was neighborhood vacancy rate, quantified as the number of vacant and abandoned homes divided by the average number of homes per CSA over the study period, then multiplied by 100. Data on vacancy rates were derived from the Baltimore Neighborhood Indicators Alliance-Jacob France Institute (BNIA-JFI) at the University of Baltimore. BNIA-JFI has collected data from a range of sources, such as government agencies, neighborhood groups, non-profit organizations, and commercial sources, and reported on over 150 neighborhood indicators at the CSA level since 2000 [37]. In particular, information on vacant and abandoned homes was originally collected by officials from the Baltimore Department of Housing and Community Development who regularly report on housing code violations in the city; homes are classified as vacant and abandoned if it is deemed not habitable. Records on all residential properties are collected and maintained by the Maryland State Department of Assessments and Taxation.

### 2.4. Confounders

Confounders were factors hypothesized to influence the independent variable, vacancy rate, and the dependent variable, food swamp index, but did not fall along the causal pathway. Neighborhood-level factors that do not vary over time are controlled for in our neighborhood-fixed effects statistical models (described below), by comparing each neighborhood to itself over time; however, factors that vary over time and might confound the relationship were included in the models. These were: (1) racial composition of a neighborhood, operationalized as the racial diversity index, which was quantified as the probability that two people selected at random in a neighborhood would be of a different race or ethnic group; (2) median sales price of homes, which served as a proxy of neighborhood socioeconomic status (SES) [38]; and (3) total population size of each neighborhood. The racial diversity index more efficiently represents changing demographics by capturing seven race groups (White, Black, American Indian, Asian, Pacific Islander, some other race, and two or more races) that can be either of Hispanic or non-Hispanic origin in one measure; however, it does not reflect which race is predominant in an area [39]. Because data on total population size and the racial diversity index were originally obtained from the decennial U.S. Censes 2000 & 2010, we used linear interpolation to estimate a continuous change in these variables for missing years.

### 2.5. Effect Measure Modifiers/Moderators

We hypothesized that the association between changes in the vacancy rate and changes in the food swamp index might vary by the following neighborhood-level factors: neighborhood SES, whether a neighborhood was predominantly African American, and neighborhood change in population size; thus, we tested interactions between these variables and our independent variable, vacancy rate. The association between vacancy rates and the food swamp index was hypothesized to vary by neighborhood SES due to differences in wealth and political power that can buffer the negative effects of neighborhood blight. For instance, interviews with residents from low-income communities of Oakland, CA revealed that high-SES neighborhoods received far more resources from the city and had their issues addressed in a much more timely manner, while in low-SES neighborhoods a supermarket was finally scheduled to open only after several years of community activism [40]. For this study, high-SES neighborhoods were defined as those with an average median sales price of homes greater than $98,000, which was the average value for median sales price of homes among CSAs over the observation period. Whether or not a CSA was predominantly African American was also hypothesized to be an effect measure modifier of the relationship between vacancy rates and the food swamp index. This was based on a hypothesis that due to institutional and individual-level racism, the institutional and storeowner response to vacant homes in predominantly African American neighborhoods may be different, compared to the analogous response in predominantly non-African American neighborhoods [41]. For our analysis, predominantly African American neighborhoods were defined as those with greater than 62 percent African American residents, based on the average proportion of African American residents across CSAs within the city. For ease of understanding, henceforth we will refer to predominantly African American neighborhoods as African American neighborhoods. Lastly, we hypothesized population change as an effect measure modifier of the relationship between vacancy rates and the food swamp index because neighborhoods experiencing depopulation likely represents an overall decline and disinvestment in a neighborhood [42], while gains in population size likely represent strengthening and development in a neighborhood. Therefore, it is reasonable that in areas with growth and perceived investment, the association between vacancy rates and the food swamp index may be less severe than in areas with the opposite trend. For this study, population change was quantified by subtracting a neighborhood’s population in 2001 from that same neighborhood’s population in 2012; a positive change indicated a gain in population size, while a negative change indicated a loss in population size over time.

### 2.6. Statistical Analysis

For our primary analyses, we used fixed effects linear regression models to determine the extent to which changes in neighborhood vacancy rates were associated with changes in the food swamp index across the 55 CSAs from 2001 to 2012. Fixed effects models are longitudinal models that can account for time-invariant, unobserved neighborhood effects by comparing CSAs to themselves over time (i.e., each CSA serves as its own control), as opposed to comparing CSAs with different levels of vacancy rates and unobserved neighborhood effects to each other. Specifically, CSA-fixed effects estimate the deviation from the neighborhood mean for each observation, thereby leaving measurement of changes in the independent variable and its net effect on changes in the dependent variable. We also evaluated the impact of a one-year lag in vacancy rates on the food swamp index, given that the effect of vacant homes may take time to manifest during the first year and then plateau thereafter [43]. Due to the change in census tract boundaries in 2010, we also conducted additional analyses by excluding CSA 52 and CSA 53 for the years 2001 through 2009.

We tested each of the 6 possible interactions (vacancy rate or lagged vacancy rate each interacted with African American neighborhoods, neighborhood SES, and population change) using the Wald test of the interaction term in separate pooled models. All models included a fixed effect for each CSA that accounted for baseline differences by CSA and indicator variables for each year that accounted for any secular trends in the outcome. Included in our models as time-varying covariates were neighborhood racial diversity index, median sales price of homes, and total population size. Statistical significance was set at 0.10 for main effects [44] and interactions [45], since fixed effects models have notoriously low power to detect effects and since interactions also have lower power than main effects [46].

To evaluate the degree of spatial autocorrelation between neighboring communities, we calculated the global Moran’s I statistic [47] for the regression residuals from our fully adjusted fixed effect models, using GeoDa 1.8.12 (School of Geographical Sciences and Urban Planning: Tempe, AZ, USA, 2016) [48]. Moran’s I values can range from −1 and 1, indicating dispersion and spatial dependence, respectively; values closer to 0 indicate spatial randomness.

### 2.7. Sensitivity Analyses

Prior studies have shown that segregation by race and income can impact the availability of food outlets in a neighborhood [41,49]. Thus, in sensitivity analyses, we tested whether the associations of interest varied when African American neighborhoods were defined as those with greater than 93% African American residents, which was the 75th percentile of the proportion of residents in each CSA who were African American. We also tested whether the associations of interest varied when high-SES neighborhoods were defined as those with an average median sales price of homes greater than $162,000 based on the distribution of the data and at the 75th percentile.

## 3. Results

**Figure 3:**
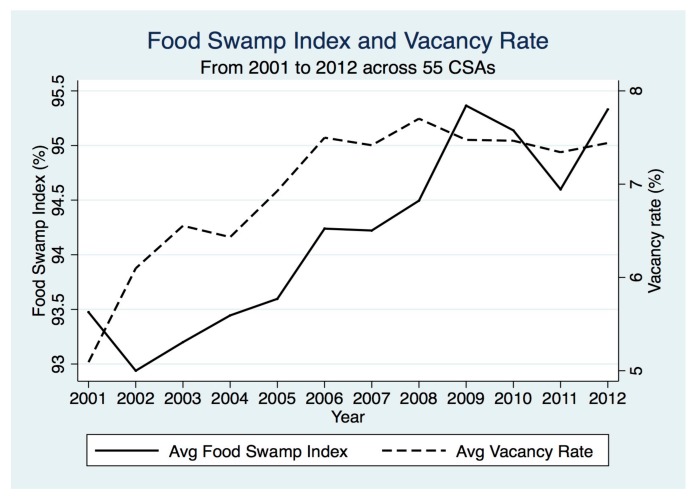
Average food swamp index and average vacancy rate across 55 CSAs in Baltimore, MD, USA, over the study period from 2001 to 2012.

Over the 12-year period, our study included 2967–3580 food outlets. By 2012, the vacancy rate in the City increased by 2.6 percentage points (Table 1). The average food swamp index across all CSAs increased by nearly 2 percentage points from 93.5 to 95.3 (Figure 3).

The density of BMI-unhealthy and BMI-intermediate outlets increased on average by 5.2 (range in change: −11.5 to 38.6) outlets per square mile and by 7.4 (range in change: −2.4 to 65.7) outlets per square mile, respectively. Conversely, the density of BMI-healthy outlets decreased on average by −0.86 (range in change: −15.7 to 12.1) outlets per square mile. Of the 31 CSAs that were classified as African American neighborhoods, eight were high-SES neighborhoods, and of the 24 CSAs that were classified as non-African American neighborhoods, 19 were high-SES neighborhoods (Table S4). The average median sales price of homes in African American neighborhoods was $82,580, compared to $166,575 in non-African American neighborhoods (not shown).

### 3.1. Association between Changes in Vacancy Rates and Changes in Food Swamps

The association between concurrent changes in vacancy rates and changes in the food swamp index varied by whether or not neighborhoods were predominantly African American (b_vacancyXAfrican American neighborhood_: 0.37; 90% CI, 0.045 to 0.69; *p*-value: 0.061) and by neighborhood SES (b_vacancy rateXSES_: −0.36; 90% CI, −0.67 to −0.047; *p*-value: 0.059), so we present results from separate models, stratified by each of these factors (Table 2). The variation by population change was not statistically significant (b_vacant rateXpopulation change_: 0.33; 90% CI, −0.0029 to 0.66; *p*-value: 0.10). Results from the exclusion of CSA 52 and CSA 53 for the years 2001 through 2009 were found to be similar as results from the main models (Table S5).

In models stratified by whether a neighborhood was predominantly African American, we found no statistically significant relationship between changes in vacancy rates and changes in the food swamp index, among African American neighborhoods. However, when comparing non-African American neighborhoods to themselves over the study period, a one-percentage point increase in concurrent vacancy rates was associated with a statistically significant decrease in the food swamp index (i.e., a shift towards a healthier food environment) (b = −0.38; 90% CI, −0.64 to −0.12; *p*-value: 0.015), after accounting for changes in neighborhood SES, racial diversity, and total population size (Table 2).

In models stratified by neighborhood SES, increases in vacancy rates were associated with increases in the unhealthiness of the food environment among poorer neighborhoods. Specifically, when comparing low-SES neighborhoods to themselves over the study period, a one-unit increase in the vacancy rate was associated with a statistically significant increase in food swamp index by 0.15 percentage points (i.e., a shift towards an unhealthier food environment) (90% CI, 0.037 to 0.27; *p*-value: 0.031), after accounting for concurrent changes in neighborhood racial diversity and total population size. For high-SES neighborhoods, the relationship between changes in concurrent vacancy rates and changes in the food swamp index was negative (i.e., trending towards a healthier food environment), but did not reach statistical significance (Table 2).

The relationship between concurrent vacancy rates and the food swamp index did not vary significantly by population change. When using the one-year lagged vacancy rate, the relationship did not vary statistically significantly by any of the effect measure modifers: neighborhood SES, whether a neighborhood population was predominantly African American, or population change. The pooled model indicated that vacancy rates lagged by one year was not statistically significantly associated with the food swamp index.

The Moran’s I value for the food swamp index was significantly positive in some years indicating spatial similarity for neighboring CSAs; however, the Moran’s I value on the regression residuals was not significantly different from zero, with the exception of 2008, indicating lack of spatial similarity for adjacent CSAs, after controlling for neighborhood racial diversity index, median sales price of homes, and total population size (Table S6).

### 3.2. Sensitivity Analyses

To assess the extent to which results would change based on how neighborhood SES and African American neighborhoods were defined, we performed sensitivity analyses with African American neighborhoods defined as those with greater than 93% African American residents and with high-SES neighborhoods defined as those with an average median sales price of homes greater than $162,000. Results were substantively the same as primary analyses when altering the high-SES cut-off point. When ‘predominantly African American’ was defined as >93% African American residents, comparing African American neighborhoods to themselves over the study period, increases in concurrent vacancy rates were associated with statistically significant increases in the food swamp index (i.e., a shift towards an unhealthier food environment) (b = 0.16; 90% CI, 0.0052 to 0.31; *p*-value: 0.089), controlling for concurrent change in neighborhood racial diversity, neighborhood SES, and total population size (Table 3). This was different from primary results for African American neighborhoods based on the 62% cut-off definition at the citywide average, in which we saw no significant association between changes in vacancy rates and changes in the food swamp index, but similar to results found among low-SES neighborhoods.

## 4. Discussion

Our study adds to the body of work on neighborhood factors related to the food environment by investigating the extent to which disrepair in the physical appearance of an area may compromise access to healthier food outlets. To our knowledge, this is the first to investigate the relationship between annual changes in vacancy rates and food swamps in an urban setting, over a 12-year period. Overall, we found that the association between vacant homes and food swamps differed by whether or not a neighborhood was predominantly African American. Contrary to expectation, among non-African American neighborhoods, we found that an increase in vacancy rates was associated with a decrease in the food swamp index (i.e., a change towards a less unhealthy environment). In addition, our results show that the relationship between changes in vacancy rates and changes in the food swamp index varied by neighborhood SES. Specifically, among poorer neighborhoods, increases in vacancy rates were associated with a more unhealthy food environment.

We reveal important differences in the impact of vacancies on food swamps across African American and non-African American neighborhoods. Based on the 93% African American cut-off in the sensitivity analysis, we observed the same positive association among African American neighborhoods found in the main analysis. However, based on the higher cut-off that further isolated the effect of a neighborhood that is ‘predominantly African American,’ the association between concurrent vacancy rates and food swamps reached statistical significance. It is also worth noting that the vast majority of African American neighborhoods (at the 93% cut-off) were low-SES, making it challenging to disentangle the separate effects of segregation by race versus poverty. Reasons for why a potentially detrimental exposure, like vacant homes, would be associated with more positive food environments in non-African American neighborhoods while associated with an increasing food swamp index in poor and African American neighborhoods remain perplexing. However, taken together, we speculate these findings point to potentially systemic disadvantages among African American communities also documented in prior literature. For instance, one study reported that the impact of the U.S. foreclosure crisis of early 2000 was unevenly distributed across racial lines, with White neighborhoods experiencing an average foreclosure rate of 2.3 percent and predominantly African American neighborhoods experiencing foreclosure rates about three time as high [50]. This unequal burden in economic and housing instability is likely also to have profound impacts on the concentration of vacant and abandoned properties in African American versus White neighborhoods. Additionally, vacant homes may not indicate the same thing in non-African American versus African American neighborhoods. That is, vacancies could indicate a neighborhood in transition or in the process of revitalization when the racial makeup is predominantly non-African American, and it could indicate stagnation or persistent disinvestment when the neighborhood is predominantly African-American. 

Researchers have also reported that, after controlling for SES, an increase in foreclosed homes reduced the presence of White residents but increased the presence of African American and Latino residents, suggesting that White families had opportunities to shield themselves from the foreclosure crisis and move out of declining neighborhoods, while African American and Latino families moved in [50]. Furthermore, while unhealthy food outlets such as fast food restaurants target both African American and White communities, the power of social capital, political influence, and financial resources have been shown to successfully stymie McDonald’s, for example, and its attempt to open in one of New York City’s predominantly White neighborhoods [41]. This difference in food availability among White neighborhoods was also paralleled in another study, describing that the proportion of a neighborhood’s population that is White was negatively associated with the density of fast food restaurants in low-income neighborhoods over time [51]. Based on these former findings, another possible explanation for the counterintuitive results in non-African American neighborhoods is that such areas may have additional resources to buffer some negative exposures and perhaps use social capital, community organizing, or advocacy to influence whether certain food outlets move into a neighborhood. To build greater understanding of these experiences in racially segregated neighborhoods, future research can consider exploring the extent to which vacant homes may impact the food environment in low- and high-income, White neighborhoods compared to low- and high-income African American neighborhoods over time.

Our results also indicate that increasing vacancies are associated with increasing food swamps among both low-SES neighborhoods (with African American and non-African American neighborhoods represented) and among African American neighborhoods (in which mainly low-SES neighborhoods were represented; all but one CSA with 93% or more African American residents were also low-SES in this context). These findings build upon and are consistent with prior examinations of food outlets in the U.S. and Canada, reporting that socioeconomically disadvantaged neighborhoods tend to be associated with greater relative access to unhealthier food outlets, such as fast food restaurants or convenience stores, compared to more advantaged neighborhoods [51,52]. Although some recent strategies aiming to increase healthy food availability in impoverished areas have relocated a farmers’ market to the city center, converted a vacant lot to an urban farm, and implemented the acceptance of federal food assistance vouchers for use at farmers’ markets [53,54,55], these examples are unique cases. The disproportionate availability of smaller outlets such as fast food restaurants, convenience, and corner stores that ‘swamp out’ healthier food outlets remains an issue in many urban communities. Furthermore, prior literature offers some explanations as to why the relationship between vacancy rates and food swamps is exacerbated in low-SES neighborhoods. As storeowners consider location characteristics, there is minimal motivation for a variety of storeowners to establish their businesses in this context. For instance, larger outlets, such as supermarkets that offer a greater variety of healthy food options at a lower price point, have few incentives pulling them to disadvantaged neighborhoods due to fears related to crime and safety, higher operation costs (e.g., rent, insurance), and declining middle-class populations [56,57,58]. Still, where there are people, there is a market, and smaller, less healthy food outlets tend to fill this need. Boarded-up vacant homes have also been reported to overshadow positive features of a neighborhood by undermining attempts to improve the image or overall success of the neighborhood [14]. Our findings elaborate on the ‘broken windows’ theory, and we postulate that this visibility of vacant homes can be applied to storeowners. While storeowners perceive a decline in the neighborhood and limited monitoring of behaviors, signaled by vacant homes and ‘broken windows’ left unfixed, certain food outlet types may be more inclined to open or stay open, while others may be more inclined to look elsewhere.

Until recently, more attention has been given to understand the behaviors of consumers given a particular food environment. Some researchers have also begun to explore the dynamic nature of food access, accounting for changing food landscapes over space and time as individuals move from one place to another [59]. Nonetheless, few studies have looked at neighborhood-level risk factors that shape the mix of food outlets in communities. We build on this existing literature by exploring the extent to which vacant homes might influence the overall composition of food outlets in urban neighborhoods, particularly focusing on the disproportionate availability of unhealthy food outlets as compared to all food outlets, including healthy ones, through the use of a food swamp index. Our modeling approach is also a unique element of this study. We performed fixed effects linear regression models, which are particularly advantageous for this type of analysis because they provide a means to reduce the effect of omitted variable bias and confounding (e.g., self-selection). Such models address this common limitation by allowing each CSA to serve as its own control over the study period (i.e., each CSA is compared to itself over time) [3]. Lastly, we improve upon previous research by demonstrating longitudinal associations between changes in vacancy rates and changes in food swamps, instead of examining the static, cross-sectional relationships.

Despite these strengths, this study has some limitations. First, there are limitations in the measurement of the food environment. There is certainly variability in foods offered within stores of the same category; however, this level of data does not exist for all food establishments. Thus, we are left with broader categorizations of unhealthy, intermediate, and healthy food outlets. Additional sources of food environment data may have included the City’s database of licensed food establishments or on-the-ground verification (i.e., ‘ground-truthing’) of food outlets, but these approaches are especially challenging for a longitudinal study of this size. Therefore, we implemented the recommended solution based on prior validation research of commercial datasets and used a combined database to improve levels of sensitivity. Further, since misclassification is a common issue in commercial datasets, we took extra steps to reduce this measurement error by tagging food outlets based on a range of keywords (e.g., carry-out, pizza, burger), therefore, ensuring consistent classification across the study period. Second, there are limitations of a neighborhood fixed effects analyses worth noting. Because fixed effects models rely on within-neighborhood changes over time in both the outcome and exposure variables, this can result in larger standard errors, leading to higher *p*-values and wider confidence intervals. In addition, the effect of time invariant variables, such as the distance of each neighborhood from the city center, cannot be estimated through the use of these models, although such characteristics at baseline are controlled for with fixed effects models. Third, we do not include vacant homes just beyond the City’s boundaries, which may potentially impact food outlets within city limits. However, the landscape and policies related to housing and food establishments in counties adjacent to the City or rural areas are different from those in urban neighborhoods, so we relied on community boundaries as defined by the City and City planners’ understanding of residents’ perceptions of community boundaries. Fourth, because data on vacant homes were previously collected for administrative reasons, they may be prone to more measurement error than if these data had been collected for research purposes. Still, other data sources such as the U.S. Postal Service and U.S. Census, which employ more liberal definitions of vacant homes, capture homes that are unoccupied but still habitable (i.e., do not violate housing code regulations), making data from Baltimore City Housing and BNIA the more appropriate and reliable option for this study [60,61]. Finally, while we report the final geocoding match rate, we do not have available the match rate before our review of matched tied addresses.

Moving forward, boarded-up vacant homes provide unique opportunities to not only improve the neighborhood environment, but acting upon them could potentially influence downstream factors, such as the composition of food outlets in an area. Additionally, many jurisdictions across the U.S. have embraced a ‘health in all policies’ standpoint in support of routine consideration of health in traditionally non-health decisions [62]. From this perspective, this is a chance to foster collaborations between the housing, public health, and other sectors to bolster knowledge- and capacity-building around how addressing vacant homes can improve the health of residents. For example, in response to the U.S. foreclosure crisis, the U.S. Conference of Mayors launched efforts to address the problem across 77 cities in 30 states, including the Vacants to Value initiative in Baltimore, MD [63]; conversion of vacant lots to green space in Philadelphia, PA [64]; and retail development as part of the Neighborhood Renaissance Savannah revitalization project in Savannah, GA [65], to name a few. Furthermore, important to consider is a likely unfair advantage of non-African American neighborhoods. Boarded-up vacant homes appear to have a more negative impact in African American neighborhoods, where residents may face a disproportionate burden with the most limited access to quality food and greatest access to unhealthy food, therefore, should be prioritized for interventions. In particular, attention should be given to eliminate segregation by race and income, so that neighborhood amenities are equitably distributed and accessible to all residents. To better inform such future efforts, follow-up research would benefit from examining different proxies of segregation, such as isolation, as determinants of food swamps and its impact in other contexts, such as rural settings. More quantitative and qualitative studies are also needed in exploring the differences in social networks and political capital of low-SES and high-SES neighborhoods, and how those differences relate to vacant homes and the neighborhood food environment.

## 5. Conclusions

Our study adds to the neighborhood food environment literature by considering the influence of boarded-up vacant homes on food swamps. We found that vacant homes may compromise the neighborhood food environment by increasing the relative density of unhealthy and intermediate food outlets out of all food outlets, or food swamp index. Most notably, in non-African American neighborhoods increasing vacancy rates were associated with statistically significant decreases in food swamps, while in poorer neighborhoods, increasing vacancy rates were associated with statistically significant increases in food swamps. Elaborating on the ‘broken windows’ theory, we speculate that vacant homes may signal neighborhood economic decline that affect not only residents’ perceptions but also the perception of other community members, such as food storeowners. Greater efforts are needed to improve neighborhood conditions and more equitably distribute sources of healthy food across communities, which also is likely to have implications related to residents’ dietary behaviors and health outcomes.

## Figures and Tables

**Table 1:** Summary statistics of key variables for community statistical areas (CSAs) (n = 55) in Baltimore, MD, in 2001 and 2012.

	2001 Mean (SD)	2012 Mean (SD)
Median sales price of homes ($)	76,462 (40,894)	114,812 (86,652)
Racial diversity index (%)	28.7 (18.8)	38.1 (23.3)
Total population (n)	11,675 (4560)	11,314 (4435)
Vacancy rate (%)	5.1 (6.9)	7.7 (10.1)
Food swamp index (%)	93.5 (6.1)	95.3 (4.6)
BMI-unhealthy outlet density (outlets/sq. mile)	39.7 (47.5)	45.0 (53.3)
BMI-intermediate outlet density (outlets/sq. mile)	14.1 (26.5)	21.5 (38.4)
BMI-healthy outlet density (outlets/sq. mile)	4.0 (7.0)	3.2 (6.4)

**Table 2:** Neighborhood fixed effects linear regression ^1^ for the relationship between changes in vacancy rates ^2^ and changes in the food swamp index ^3^ in Baltimore, MD, over the study period from 2001 to 2012. Results are from the pooled model and stratified models by African American neighborhoods and neighborhood SES.

	Food Swamp Index									
	Pooled Model (n = 55)		Stratified Models (by Predominantly African American ^4^)				Stratified Models (by Neighborhood SES ^5^)			
			Non-African American ≤ 62% (n = 24)		African American > 62% (n = 31)		High-SES > $98,000 (n = 27)		Low-SES ≤ $98,000 (n = 28)	
	b (90% CI)	*p*-value	b (90% CI)	*p*-value	b (90% CI)	*p*-value	b (90% CI)	*p*-value	b (90% CI)	*p*-value
Vacancy rate	0.041 (−0.095, 0.18)	0.62	−0.38 * (−0.64, −0.12)	0.015	0.078 (−0.11, 0.27)	0.50	−0.23 (−0.57, 0.11)	0.26	0.15 (0.037, 0.27)	0.031
Vacancy rate, 1-year lag	−0.024 (−0.17, 0.12)	0.79	-				-			

^1^ Models for the food swamp index is a neighborhood fixed effects linear regression model. Models include a fixed effect for each CSA, which accounts for baseline differences by CSA, indicator variables for each year to account for any secular trends in the outcome, and as time-varying covariates, neighborhood racial diversity index, median sales price of homes, and total population size. In the pooled model, we tested separately the interaction for vacancy rate and African American neighborhoods, for vacancy rate and neighborhood SES, and for vacancy rate and population change. The association between changes in concurrent vacancy rates and changes in the food swamp index varied by whether a neighborhood was predominantly African American (b_vacancy rateXAfrican American neighborhood_: 0.37; 90% CI, 0.045 to 0.69; *p*-value: 0.061) and by neighborhood SES (b_vacancy rateXSES_: −0.36; 90% CI, −0.67 to −0.047; *p*-value: 0.059), so we present results from separate models. The variation in the association between vacancy rate and the food swamp index by population change did not reach statistical significance (b_vacancy rateXpopulation change_: 0.33; 90% CI, −0.0029 to 0.66; *p*-value: 0.10). The association between changes in vacancy rates lagged by one year and changes in the food swamp index also did not statistically significantly vary by whether a neighborhood was predominantly African American (b_lagged vacancy rateXAfrican American neighborhood_: 0.30; 90% CI,−0.047 to 0.64; *p*-value: 0.16), by neighborhood SES (b_lagged vacancy rateXSES_: −0.19, 90% CI, −0.53 to 0.15; *p*-value: 0.36) or by population change (b_lagged vacancy rateXpopulation change_: 0.30; 90% CI, −0.054 to 0.66; *p*-value: 0.16). ^2^ Vacancy rate was defined as the number of vacant and abandoned homes divided by the average number of homes, then multiplied by 100. Homes are classified as vacant and abandoned by Baltimore City Housing if the property is not habitable. ^3^ Food swamp index was defined as the density (outlets per sq. mile) of BMI-unhealthy and BMI-intermediate outlets out of the density of all food outlets, including BMI-healthy outlets, then multiplied by 100. This index was an adaptation of the Modified Retail Food Environment Index (mRFEI) [28] and Physical Food Environment Index (PFEI) [29]. ^4^ Predominantly African American neighborhoods were defined as those with greater than 62 percent African American residents, based on the City’s average proportion of African American residents across CSAs over the observation period. ^5^ High-SES neighborhoods were defined as those with an average median sales price of homes greater than $98,000, which was the average value for median sales price among CSAs over the observation period. * Interpretation: On average, when comparing non-African American neighborhoods to themselves over the study period, for each percentage point increase in the vacancy rate, there was a statistically significant decrease in the food swamp index by −0.38 percentage points (90% CI, −0.64 to −0.12; *p*-value: 0.015), controlling for neighborhood racial diversity index, median sales price of homes, total population size, and baseline time-invariant neighborhood characteristics.

**Table 3:** Pooled model and sensitivity analyses ^1^, testing whether the relationship between changes in vacancy rates ^2^ and changes in the food swamp index ^3^ differs when varying the definition for predominantly African American neighborhoods ^4^ and for neighborhood SES ^5^.

	Food Swamp Index ^5^									
	Pooled Model (n = 55)		Stratified Models (by Predominantly African American)				Stratified Models (by Neighborhood SES)			
			Non-African American ≤ 93% (n = 41)		African American > 93% (n = 14)		High-SES > $162,000 (n = 13)		Low-SES ≤ $162,000 (n = 42)	
	b (90% CI)	*p*-value	b (90% CI)	*p*-value	b (90% CI)	*p*-value	b (90% CI)	*p*-value	b (90% CI)	*p*-value
Vacancy rate	0.041 (−0.095, 0.18)	0.62	0.040 (−0.17, 0.25)	0.75	0.16 (0.0052, 0.31)	0.089	−0.33 (−0.82, 0.16)	0.27	0.13 (0.012, 0.24)	0.069

^1^ The statistical models for the food swamp index is a neighborhood fixed effects linear regression model, which includes a fixed effect for each CSA (accounting for baseline differences by CSA), indicator variables for each year (accounting for any secular trends in the outcome), and as time-varying covariates, neighborhood racial diversity index, median sales price of homes, and total population size. ^2^ Vacancy rate was defined as the number of vacant and abandoned homes divided by the average number of homes, then multiplied by 100. Homes are classified as vacant and abandoned by Baltimore City Housing if the property is not habitable. ^3^ Food swamp index was defined as the density (outlets per sq. mile) of BMI-unhealthy and BMI-intermediate outlets out of the density of all food outlets, including BMI-healthy outlets, then multiplied by 100. This index was an adaptation of the Modified Retail Food Environment Index (mRFEI) [28] and Physical Food Environment Index (PFEI) [29]. ^4^ Predominantly African American neighborhoods were defined as those with greater than 93% African American residents, which was the 75th percentile of the proportion of residents in each CSA who were African American across CSAs over the observation period. ^5^ High-SES neighborhoods were defined as those with an average median sales price of homes greater than $162,000 based on the distribution of the data and at the 75th percentile.

## Supplementary Materials

The following are available online at www.mdpi.com/1660-4601/14/11/1426/s1, Table S1: Each of 175 SIC definitions classified into one of 10 crude food outlet categories. Table S2: Keywords used to identify and tag dollar stores and carry-out restaurants. Table S3: Top 50 fast food chain restaurants in the U.S, reported by the leading source of information for the quick-service industry, QRS Magazine (Available at: https://www.qsrmagazine.com/reports/qsr50-2015-top-50-chart). Table S4: Average median sales price of homes of CSAs, according to neighborhood SES and proportion of the neighborhood population that is African American. Table S5: Neighborhood fixed effects linear regression for the relationship between changes in vacancy rates and changes in the food swamp index in Baltimore, MD, over the study period from 2001 to 2012, excluding CSA 52 and CSA 53 from 2001 through 2009. Results are from the pooled model and stratified models by African American neighborhoods and neighborhood SES. Table S6: Global Moran’s I statistic for the regression residuals, over the study period.
