# Supplementary material for: Relationships between Vacant Homes and Food Swamps: A Longitudinal Study of an Urban Food Environment
Source: Int J Environ Res Public Health. 2017 Nov 21;14(11):1426. doi: 10.3390/ijerph14111426 (PMC5708065; doi:10.3390/ijerph14111426)
Supplement: Supplementary file 1 [file ijerph-14-01426-s001.pdf]

Supplementary Materials

Table S1. Each of 175 SIC definitions classified into one of 10 crude food outlet categories [17].

SIC definition	Crude food outlet category
1. Beverage stores	Carry-out restaurants
2. Box lunch stand	
3. Cafe	
4. Cafes	
5. Cafeteria	
6. Carry-out only (except pizza) restaurant	
7. Chicken restaurant	
8. Chili stand	
9. Coffee	
10. Coffee & tea	
11. Coffee roasting & handling equipment	
12. Coffee shop	
13. Coffee shops	
14. Delicatessen (eating places)	Carry-out restaurants cont.
15. Delicatessen stores	
16. Delicatessens	
17. Drive-in restaurant	
18. Food bars	
19. Foods-carry out	
20. Hamburger stand	
21. Health food restaurant	
22. Hot dog stand	
23. Luncheonette	
24. Lunchrooms and cafeterias	
25. Pizza	
26. Pizza restaurants	
27. Pizzeria, chain	
28. Pizzeria, independent	
29. Sandwiches	
30. Sandwiches and submarines shop	
31. Tea	
32. Tea rooms	
33. Restaurants-food delivery	
34. Convenience stores	Convenience stores
35. Convenience stores, chain	
36. Convenience stores, independent	
37. Dairy products stores	
38. Dairy products-retail	
39. Filling stations, gasoline	
40. Gasoline service stations	
41. Petroleum products (whls)	
42. Service stations-gasoline & oil	
43. Fast food restaurants and stands	Fast-food restaurants
44. Fast-food restaurant, chain	
45. Fast-food restaurant, independent	
46. American restaurant	Full-service restaurants
47. Barbecue restaurant	
48. Buffet (eating places)	
49. Cajun restaurant	
50. Caterers	
51. Chinese restaurant	
52. Diner	
53. Eating places	
54. Ethnic food restaurants	
55. Family restaurants	
56. French restaurant	
57. Greek restaurant	
58. Grills (eating places)	
59. Indian pakistan restaurant	

60. Italian restaurant<br>61. Japanese restaurant<br>62. Korean restaurant<br>63. Macaroni spaghetti & noodles (mfrs)<br>64. Mexican restaurant<br>65. Oyster bar<br>66. Restaurant management<br>67. Restaurant, family: chain<br>68. Restaurant, family: independent<br>69. Restaurant, lunch counter<br>70. Restaurants<br>71. Seafood restaurants<br>72. Seafood shack<br>73. Spanish restaurant<br>74. Steak and barbecue restaurants<br>75. Steak restaurant<br>76. Sushi bar<br>77. Thai restaurant<br>78. Vietnamese restaurant	Full-service restaurants cont.
79. Department stores<br>80. Department stores, discount<br>81. Drug stores<br>82. Drug stores and proprietary stores<br>83. Miscellaneous general merchandise stores<br>84. Pharmacies<br>85. Proprietary (non-prescription medicine) stores<br>86. Retail shops<br>87. Variety stores	General merchandise stores
88. Dried fruit<br>89. Eggs and poultry<br>90. Eggs-retail<br>91. Fish and seafood markets<br>92. Fish markets<br>93. Food and freezer plans, meat<br>94. Food brokers (whls)<br>95. Food products & manufacturers<br>96. Frozen food and freezer plans, except meat<br>97. Fruit and vegetable markets<br>98. Fruits & vegetables & produce-retail<br>99. Health & diet foods-retail<br>100. Health and dietetic food stores<br>101. Health foods<br>102. Meat and fish markets<br>103. Meat markets-kosher<br>104. Meat markets, including freezer provisioners<br>105. Meat-retail<br>106. Meat-wholesale<br>107. Mushrooms<br>108. Nuts<br>109. Nuts-edible<br>110. Poultry-retail<br>111. Salads (whls)<br>112. Seafood markets<br>113. Seafood packers (mfrs)<br>114. Seafood-retail<br>115. Vegetable stands or markets	Healthy specialty stores<br>Healthy specialty stores cont.
116. Food products (whls)<br>117. Chinese food products<br>118. Freezer provisioners, meat<br>119. Gourmet food stores<br>120. Gourmet shops<br>121. Juices-retail<br>122. Kosher food products-wholesale<br>123. Oriental goods	Mixed specialty stores
124. Food markets<br>125. Grocers-retail	Small grocers/corner stores

126. Grocers-wholesale<br>127. Grocery stores<br>128. Grocery stores, chain<br>129. Grocery stores, independent<br>130. Miscellaneous food stores<br>131. Food products-retail<br>132. Supermarkets<br>133. Supermarkets, chain<br>134. Supermarkets, independent	
135. Wholesale clubs<br>136. Wholesalers	Superstores
137. Bagels<br>138. Bakers-retail<br>139. Bakers-wholesale<br>140. Boutique items-retail<br>141. Bread<br>142. Bread/other bakery prod-ex cookies<br>143. Butter<br>144. Cakes<br>145. Candy<br>146. Candy & confectionery-manufacturers<br>147. Candy & confectionery-retail<br>148. Candy, nut, and confectionery stores<br>149. Cheese<br>150. Chocolate & cocoa-manufacturers<br>151. Cookies<br>152. Cookies & crackers<br>153. Doughnuts<br>154. Frozen yogurt stand<br>155. Ice cream & frozen desserts-distr (whls)<br>156. Ice cream (packaged)<br>157. Ice cream parlors<br>158. Ice cream stands or dairy bars<br>159. Ice cream, soft drink and soda fountain stands	Unhealthy specialty stores
160. Italian food products<br>161. Meat products (mfrs)<br>162. Pastries<br>163. Popcorn & popcorn supplies<br>164. Pretzels<br>165. Pretzels-retail<br>166. Retail bakeries<br>167. Sausages<br>168. Sausages/other prepared meat prod (mfrs)<br>169. Snack bar<br>170. Snack products<br>171. Snack shop<br>172. Snow cone stand<br>173. Soda fountain<br>174. Soda fountain shops<br>175. Yogurt	Unhealthy specialty stores cont.

Table S2. Keywords used to identify and tag dollar stores and carry-out restaurants [17].

Keywords for dollar stores:
Dollar
Local experts also identified and previously validated the following:
Deals
J H Wholesale
Liberty Buys

Keywords for carry-out restaurants:
Chicken
Pizza
Pizzeria
Burger
Hamburgers
Chinese
China
Taco
Tacos
Deli
Delicatessen
Bagel
Bagels
Sandwich
Sandwiches
Coffee
Wings
Carry
Carry-out
Carryout

Table S3. Top 50 fast food chain restaurants in the U.S, reported by the leading source of information for the quick-service industry, QRS Magazine (Available at: https://www.qsrmagazine.com/reports/qsr50-2015-top-50-chart) [17].

Rank	Company/Chain Name	Rank	Company/Chain Name
1	McDonald’s	41	Boston Market
2	Starbucks	42	In-n-Out Burger
3	Subway	43	Moe's Southwest Grill
4	Burger King	44	Long John Silver's
5	Wendy's	45	Firehouse Subs
6	Taco Bell	46	Baskin-Robbins
7	Dunkin' Donuts	47	Jersey Mike's Subs
8	Chick-fil-A	48	Jamba Juice
9	Pizza Hut	49	Captain D's
10	Panera Bread	50	Einstein Bros. Bagels
11	KFC		
12	Domino's		
13	Sonic Drive-In		
14	Chipotle		
15	Carl's Jr./Hardee's		
16	Little Caesars		
17	Dairy Queen		
18	Arby's		
19	Jack in the Box		
20	Papa John's		
21	Popeyes Louisiana Kitchen		
22	Panda Express		
23	Whataburger		
24	Jimmy John's		
25	Zaxby's		
26	Five Guys		
27	Culver's		
28	Bojangles'		
29	Steak 'n Shake		
30	Church's Chicken		
31	Papa Murphy's		
32	El Pollo Loco		
33	Checkers/Rally's		
34	Wingstop		
35	Qdoba		
36	Krispy Kreme		
37	Del Taco		
38	White Castle		

39	Tim Hortons
40	Jason's Deli

Table S4. Average median sales price of homes of CSAs, according to neighborhood SES and proportion of the neighborhood population that is African American.

	**Predominantly African American, Cutoff at City's Average**			
	African American neighborhoods > 62%		Non-African American neighborhoods ≤ 62%	
**Neighborhood SES, cutoff at 50th percentile**	Average median sales price of homes ($)	CSA (n)	Average median sales price of homes ($)	CSA (n)
Low-SES (≤$98K)	$ 65,479	23	$ 86,749	5
High-SES (>$98K)	$ 131,744	8	$ 187,5812	19
**Neighborhood SES, cutoff at 75th percentile**	Average median sales price of homes ($)	CSA (n)	Average median sales price of homes ($)	CSA (n)
Low-SES (≤$162K)	$ 75,596	29	$ 114,564	13
High-SES (>$162K)	$ 183,847	2	$ 228,042	11
	**Predominantly African American, Cutoff at 75th Percentile**			
	African American neighborhoods > 93%		Non-African American neighborhoods ≤ 93%	
**Neighborhood SES, cutoff at 50th percentile**	Average median sales price of homes ($)	CSA (n)	Average median sales price of homes ($)	CSA (n)
Low-SES (≤$98K)	$ 60,705	13	$ 76,706	15
High-SES (>$98K)	$ 110,533	1	$ 173,364	26
**Neighborhood SES, cutoff at 75th percentile**	Average median sales price of homes ($)	CSA (n)	Average median sales price of homes ($)	CSA (n)
Low-SES (≤$162K)	$ 64,265	14	$ 99,354	28
High-SES (>$162K)	N/A	0	$ 221,243	13

The bold is to help differentiate the different subcategories/cutoff points i.e., 50th percentile cutoff and 75th percentile cutoff.

Table S5. Neighborhood fixed effects linear regression [1] for the relationship between changes in vacancy rates [2] and changes in the food swamp index [3] in Baltimore, MD, over the study period from 2001 to 2012, excluding CSA 52 and CSA 53 from 2001 through 2009. Results are from the pooled model and stratified models by African American neighborhoods and neighborhood SES.

	Food Swamp Index									
	Pooled Model		Stratified Models (By Predominantly African American [4])				Stratified Models (By Neighborhood SES [5])			
			Non-African American ≤ 62%		African American > 62%		High-SES > $98,000		Low-SES ≤$98,000	
	b (90% CI)	*p*-value	b (90% CI)	*p*-value	b (90% CI)	*p*-value	b (90% CI)	*p*-value	b (90% CI)	*p*-value
Vacancy rate	0.070 (−0.073, 0.21)	0.42	−0.38 (−0.64, −0.12)	0.015	0.15 (−0.060, 0.36)	0.24	−0.37 (−0.75, 0.021)	0.12	0.17 (0.045, 0.29)	0.025
Vacancy rate, 1-year lag	0.0031 (−0.15, 0.16)	0.97	–				–			

[1] The statistical models for the food swamp index is a neighborhood fixed effects linear regression model. Models include a fixed effect for each CSA, which accounts for baseline differences by CSA, indicator variables for each year to account for any secular trends in the outcome, and as time-varying covariates, neighborhood racial diversity index, median sales price of homes, and total population size.

[2] Vacancy rate was defined as the number of vacant and abandoned homes divided by the average number of homes, then multiplied by 100. Homes are classified as vacant and abandoned by Baltimore City Housing if the property is not habitable.

[3] Food swamp index was defined as the density (outlets per sq. mile) of BMI-unhealthy and BMI-intermediate outlets out of the density of all food outlets, including BMI-healthy outlets, then multiplied by 100. This index was an adaptation of the Modified Retail Food Environment Index (mRFEI) [28] and Physical Food Environment Index (PFEI) [29].

[4] Predominantly African American neighborhoods were defined as those with greater than 62 percent African American residents, based on the City's average proportion of African American residents across CSAs over the observation period.

[5] High-SES neighborhoods were defined as those with an average median sales price of homes greater than $98,000, which was the average value for median sales price among CSAs over the observation period.

Table S6. Global Moran's I [1] statistic for the regression residuals [2], over the study period.

Year	Moran's I (Regression Residuals)	*p*-value
2001	0.087	0.099
2002	0.062	0.110
2003	0.094	0.074
2004	0.025	0.250
2005	0.092	0.072
2006	0.110	0.061
2007	0.016	0.250
2008	0.140	0.006
2009	0.043	0.220
2010	0.075	0.120
2011	0.097	0.073
2012	0.085	0.083

[1] Moran's I is one of the most commonly used measures of global spatial autocorrelation. Values can range from −1 and 1, indicating dispersion and spatial dependence, respectively; values closer to 0 indicate spatial randomness. The null hypothesis for these significance tests is that there is no spatial autocorrelation.
[2] Residuals were calculated from our neighborhood fixed-effects linear regression models, with the primary exposure variable, vacancy rate. All models included a fixed-effect for each CSA (accounting for baseline differences by CSA), indicator variables for each year (accounting for any secular trends in the outcome), and as time-varying covariates, neighborhood racial diversity index, median sales price of homes, and total population size.
